# The Leadership Civility Framework: Advancing Leadership Theory to Cultivate Healthy Work Environments

**DOI:** 10.1155/jonm/4699241

**Published:** 2026-10-27

**Authors:** Cynthia M. Clark

**Affiliations:** ^1^ Boise State University 1910 University Drive, Norco Building Boise 83725 Idaho USA boisestate.edu

## Abstract

**Aim:**

To introduce the Leadership Civility Framework (LCF), an integrative model examining how leaders may cultivate healthy work environments (HWEs) through intentional leadership practices.

**Background:**

Nurse leaders must navigate increasing workplace complexity, workforce stress, and concerns about workplace culture while fostering civility, respect, and trust. While civility is recognized as essential to healthy organizations, an integrated framework explaining how leaders translate leadership civility into organizational influence has been lacking.

**Evaluation:**

The LCF synthesizes civility scholarship with authentic, resonant, and transformational leadership theories and conceptualizes leadership as two complementary constructs: leadership civility (who the leader is) and civility leadership (what the leader does). Five interdependent leadership competency domains serve as the developmental and behavioral bridge through which leaders translate individual competencies into actions that may contribute to healthy work cultures.

**Key Issue:**

The framework positions civility as a foundational nursing leadership competency that may contribute to psychological safety, trust, belonging, collaboration, accountability, and organizational effectiveness.

**Conclusion:**

The LCF offers a theoretically grounded model for examining how nurse leaders may cultivate HWEs through intentional leadership practices and provides propositions for future empirical investigation of workforce and organizational outcomes.

**Implications for Nursing Management:**

The framework provides nurse leaders with a foundation for leadership development, organizational culture initiatives, and future research to strengthen HWEs and workforce sustainability.

## 1. Introduction

In an era defined by rapid organizational transformation, heightened workforce polarization, and escalating occupational stress, the cultivation of workplace civility has emerged as a critical determinant of organizational effectiveness and workforce sustainability. Workplace incivility and other adverse workplace behaviors range from subtle acts of disrespect, including dismissive communication, rude nonverbal gestures, and exclusionary practices, to overt bullying, threats, and workplace violence [1]. Workplace incivility has been consistently associated with burnout, psychological distress, reduced job satisfaction, diminished employee engagement, and increased turnover intentions [2–6].

Beyond these individual consequences, uncivil work environments undermine psychological safety, limiting employees’ willingness to speak up, learn, collaborate, and raise patient safety concerns [7]. The consequences of uncivil work environments extend beyond employee well‐being to organizational performance and financial sustainability. Workplace incivility within healthcare settings has been linked to increased medical errors, compromised patient safety, poorer quality of care, adverse patient outcomes, nurse burnout, turnover intentions, and reduced organizational commitment, all of which contribute to substantial organizational and economic costs [4, 5, 8]. Workplace incivility also erodes teamwork, communication, information sharing, and psychological safety, thereby weakening the collaborative relationships essential for safe, high‐quality care [7, 8]. More broadly, employee disengagement and chronic burnout cost US employers billions of dollars annually through lost productivity, absenteeism, reduced work performance, and increased healthcare‐related expenditures [9]. Collectively, this growing body of evidence underscores that fostering respectful and psychologically safe work environments is not only an ethical responsibility but also a strategic organizational imperative [8, 9].

Although leadership is widely recognized as a primary influence on organizational culture, an important conceptual gap remains. Existing leadership models describe leader characteristics and behaviors, while workplace civility measures, such as the Workplace Civility Index, assess employees’ perceptions of interpersonal interactions [10]. However, the literature remains limited in explaining how leadership competencies are translated into intentional civility practices and how these practices contribute to healthy organizational cultures. Addressing this gap is increasingly important as organizations seek practical approaches for developing leaders capable of strengthening workplace relationships, promoting psychological safety, and sustaining cultures of civility and respect.

To address this gap, this article introduces the Leadership Civility Framework (LCF), an integrative conceptual model that provides a theoretical basis for examining how leaders translate personal values, character, and competencies into intentional leadership practices that cultivate healthy work environments (HWEs). The framework integrates contemporary leadership theory with civility scholarship and distinguishes between leadership civility, which reflects who leaders are, and civility leadership, which reflects what leaders intentionally do to shape organizational culture and foster HWEs.

The LCF emerged from a broader program of civility scholarship spanning more than 2 decades and builds conceptually on the Middle‐Range Theory of Civility in Academic Nursing (TCiAN) [11]. Whereas TCiAN explains the importance of civility and identifies leadership as a pervasive contextual influence affecting HWEs, the LCF extends that theoretical foundation by explaining how leaders operationalize civility through intentional leadership competencies and behaviors to shape organizational culture. Together, the two frameworks provide complementary explanations of why civility matters and how leaders cultivate HWEs.

## 2. Framework Development

The LCF was developed through an integrative conceptual synthesis of more than 2 decades of civility scholarship, contemporary leadership literature, and prior theoretical work on civility and HWEs. Literature was selected for its relevance to workplace civility, leadership, relationships, psychological safety, and HWEs. Authentic, resonant, and transformational leadership theories were selected because they offer complementary perspectives on the intrapersonal, relational, and organizational dimensions of leadership. Relevant concepts were iteratively compared and integrated across these perspectives, resulting in five interdependent competency domains: emotional intelligence and self‐regulation; respectful and effective communication; ethical conduct and integrity; collaboration and empowerment; and relationship building and professional growth. These domains serve as the developmental and behavioral bridge between leadership civility and civility leadership. Theoretical propositions were subsequently derived from the conceptual relationships among leadership civility, the competency domains, civility leadership, HWEs, and organizational outcomes to guide future empirical testing.

## 3. Theoretical Foundations of the LCF: Integrating Three Leadership Theories

Authentic, resonant, and transformational leadership function as complementary dimensions rather than independent approaches. Authentic leadership explains who the leader is by emphasizing character, self‐awareness, and ethical integrity. Resonant leadership explains how leaders build trusting relationships through emotional intelligence, empathy, and psychological safety. Transformational leadership explains how leaders strategically influence teams, organizations, and systems to cultivate HWEs. Together, these complementary perspectives explain how leaders translate internal capacities into organizational actions that strengthen workplace culture and organizational outcomes (Table 1).

**TABLE 1:** Theoretical foundations of the leadership civility framework.

Theory	Primary focus	Contribution to the LCF
Authentic leadership	Intrapersonal foundation	Provides the individual foundation for leadership civility.
Resonant leadership	Relational mechanism	Serves as the relational mechanism through which leadership civility becomes civility leadership.
Transformational leadership	Strategic organizational influence	Demonstrates how civility leadership shapes organizational culture through strategic action.

### 3.1. Authentic Leadership: The Individual Foundation

Authentic leadership provides the intrapersonal foundation of the LCF. Its emphasis on self‐awareness, relational transparency, internalized moral perspective, and balanced processing aligns with the character, values, and ethical judgment that define leadership civility [12–14]. These internal capacities enable leaders to respond with integrity, fairness, emotional regulation, and respect, establishing the individual foundation upon which civility leadership is built.

### 3.2. Resonant Leadership: The Relational Mechanism

Within the LCF, resonant leadership serves as the relational mechanism through which leadership civility is translated into civility leadership. Emotional intelligence, empathy, and relationship management enable leaders to transform individual competencies into relational behaviors that may cultivate trust, psychological safety, and meaningful engagement [15–17]. These relational behaviors also promote psychological empowerment, work engagement, and improved job performance among nurses [18]. Collectively, these relational processes strengthen collaboration and employee voice, enabling leaders to shape organizational culture through everyday interactions.

At the individual level, resonant leadership strengthens leadership civility by enhancing a nurse leader’s self‐awareness, emotional regulation, empathy, and respectful communication. When enacted intentionally, these competencies extend to the department and organizational levels by protecting frontline employee voice (e.g., supporting a novice nurse who raises a patient safety concern), fostering psychological safety and collaboration, and creating conditions that enable collective flourishing [19]. Together, these relational processes demonstrate how emotionally intelligent leadership translates leadership civility into civility leadership.

### 3.3. Transformational Leadership: Strategic Organizational Influence

Transformational leadership explains how leadership civility extends beyond individual interactions to shape organizational culture. Within the LCF, it reflects the strategic application of civility leadership through vision, empowerment, and organizational influence. This conceptualization closely aligns with the Magnet model’s emphasis on transformational leadership and structural empowerment (American Nurses Credentialing Center [20]), whereby nurse leaders cultivate professional practice environments characterized by psychological safety, shared decision‐making, and excellence in patient care. Leaders establish clear behavioral expectations, reinforce accountability, promote inclusion, and foster cultures of trust, collaboration, and continuous improvement. In doing so, transformational leadership illustrates how leadership civility may be translated into practices that contribute to HWEs, workforce well‐being, and organizational effectiveness.

Taken together, the authentic, resonant, and transformational leadership theories provide a theoretical foundation for the LCF by explaining how leaders translate individual competencies into relational influence and strategic organizational action. Through the five leadership competency domains, leadership civility becomes civility leadership, enabling leaders to cultivate HWEs characterized by psychological safety, trust, belonging, collaboration, accountability, and organizational effectiveness. Table 1 compares the primary focus of each leadership theory and its contribution to the LCF.

These complementary theoretical perspectives establish the conceptual foundation of the LCF and provide a basis for examining its implications for leadership development, research, and nursing practice.

## 4. Core Constructs, Five Competency Domains, and Level of Analysis

Within the LCF, leadership is conceptualized across distinct but interconnected levels of analysis: individual capacity, developmental mechanisms, and organizational enactment. Leadership civility represents the individual‐level foundation (who the leader is). It encompasses the leader’s character, values, dispositions, and cognitive, affective, behavioral, and ethical capacities that shape how they think, communicate, make decisions, relate to others, exercise authority, and respond to challenges. These foundational capacities may support leaders in preserving human dignity, building trust, fostering psychological safety, and creating conditions for individual, team, and organizational effectiveness.

The five leadership competency domains represent individual‐level competencies that translate internal capacities into observable capabilities: (1) emotional intelligence and self‐regulation, (2) respectful and effective communication, (3) ethical conduct and integrity, (4) collaboration and empowerment, and (5) relationship building and professional growth. Rather than functioning as isolated attributes, these domains serve as the developmental and behavioral bridge through which leadership civility is translated into deliberate leadership practices.

Civility leadership represents the intentional enactment of these competencies through behaviors and relational practices that influence individuals, teams, and organizational culture (what the leader intentionally does). It encompasses the deliberate actions leaders take to establish behavioral expectations, cultivate psychologically safe environments, foster collaboration, address uncivil behaviors constructively, support employee well‐being, and improve organizational outcomes [1, 21]. For example, nurse leaders operationalize civility leadership and foster psychological safety during unit staff meetings, shared governance councils, debriefings following adverse clinical events, and one‐on‐one coaching conversations by actively inviting diverse perspectives, encouraging respectful dialog, and responding constructively to staff concerns. Together, these complementary constructs explain how leaders translate individual competencies into sustained organizational influence that cultivates HWEs.

## 5. Organizational Context and Framework Boundaries

The LCF recognizes that leadership practices occur within organizational contexts that can enable or constrain their enactment and effects. Staffing levels, workload, available resources, organizational policies, governance structures, leadership support, power dynamics, and communication systems may influence leaders’ capacity to enact civility competencies and may strengthen or attenuate the relationships proposed in the framework. Thus, the LCF does not assume that leadership civility or civility leadership alone determines HWEs. Rather, organizational conditions provide a contextual boundary within which leadership competencies are enacted and may shape the extent to which intentional civility practices influence psychological safety, trust, inclusion, collaboration, engagement, and HWE outcomes.

The framework is bound primarily to leadership within nursing and healthcare organizations and is intended to explain how leaders translate individual civility competencies into intentional leadership practices within complex organizational environments. It is not intended to represent all determinants of HWEs or to imply direct causation among all framework components. Because the LCF is conceptual and has not yet been empirically tested, the nature and strength of these contextual influences remain to be determined through future research.

## 6. The LCF

The LCF integrated model synthesizes these theoretical foundations, core constructs, and contextual conditions. Figure 1 illustrates the LCF and the dynamic relationships among leadership civility, the five leadership competency domains, civility leadership, HWEs, and organizational outcomes.

**FIGURE 1:**
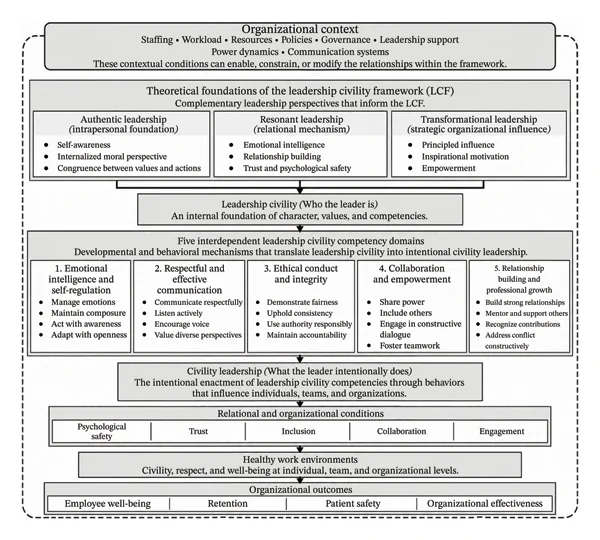
The leadership civility framework.

Five interdependent leadership competency domains serve as the developmental and behavioral bridge through which leadership civility is translated into civility leadership, with proposed implications for HWEs and organizational outcomes. Table 2 provides illustrative examples of civility leadership behaviors across the five leadership competency domains.

**TABLE 2:** Illustrative examples of civility leadership behaviors in nursing.

Competency domain	Illustrative leader behaviors
Emotional intelligence and self‐regulation	Remains calm during staffing crises; models respectful responses under pressure; seeks feedback and reflects on leadership behaviors.
Respectful and effective communication	Facilitates psychologically safe meetings; actively solicits diverse viewpoints through participatory governance; ensures every team member has a voice.
Ethical conduct and integrity	Makes transparent staffing and budgeting decisions; maintains confidentiality; addresses disrespectful behavior consistently regardless of role or seniority.
Collaboration and empowerment	Empowers shared decision‐making; supports participatory governance; encourages interprofessional collaboration and innovation.
Relationship building and professional growth	Coaches staff following challenging events; mentors others; recognizes achievements; supports professional development and career advancement.

The five competency domains were selected because they represent distinct yet complementary dimensions of leadership civility identified across the civility literature and the three leadership perspectives. The domains reflect different primary areas of leadership functioning: emotional intelligence and self‐regulation addresses intrapersonal awareness and regulation; respectful and effective communication addresses how leaders communicate and facilitate voice; ethical conduct and integrity addresses values, fairness, accountability, and responsible use of authority; collaboration and empowerment addresses shared decision‐making, participation, and agency; and relationship building and professional growth addresses mentoring, recognition, support, and development. Although some behaviors may reflect more than one domain, classification is based on the behavior’s primary purpose and underlying competency, recognizing that the domains are interdependent rather than mutually exclusive.

## 7. Theoretical Propositions

Building on the conceptual relationships illustrated in Figure 1 and the competency domains described in Table 2, five theoretical propositions are advanced to guide future empirical investigation. Within the LCF, leadership civility functions as the individual‐level antecedent; the five competency domains function as developmental and behavioral mechanisms; and civility leadership represents the intentional enactment through which leadership civility may influence relational and organizational conditions. The five domains are interdependent rather than sequential; leaders may draw on multiple domains simultaneously when enacting civility leadership. The proposed relationships are directional and are intended to be empirically testable through assessment of associations among leadership civility, competency domains, civility leadership, and HWE outcomes. These relationships are expected to operate within organizational contexts that may enable, constrain, or modify the enactment and effects of civility leadership.

 Proposition 1. Leadership civility positively influences civility leadership.
 Proposition 2. The five leadership competency domains—emotional intelligence and self‐regulation; respectful and effective communication; ethical conduct and integrity; collaboration and empowerment; and relationship building and professional growth—mediate the relationship between leadership civility and civility leadership.
 Proposition 3. Civility leadership is expected to positively influence psychological safety, trust, inclusion, collaboration, and engagement within organizations.
 Proposition 4. Civility leadership is expected to positively influence HWEs through its effects on psychological safety, trust, inclusion, collaboration, and engagement.
 Proposition 5. Leadership development interventions that strengthen leadership civility competencies are expected to increase civility leadership and contribute to healthier organizational cultures.

## 8. Discussion

The LCF extends existing civility science by providing a leadership framework that proposes how leaders may cultivate HWEs through intentional civility practices. Building on the TCiAN [11], in which leadership emerged as a pervasive contextual influence on workplace civility, the LCF proposes how leaders operationalize civility by integrating individual competencies, relational processes, and strategic organizational action. These complementary frameworks advance understanding of both why civility is essential and how leaders foster cultures characterized by civility and respect.

A distinguishing contribution of the LCF is its conceptual differentiation between leadership civility and civility leadership and its identification of five interdependent competency domains that explain how leaders operationalize civility in everyday practice. These domains function as the developmental and behavioral bridge that translates leadership civility into civility leadership. Rather than functioning as isolated leadership attributes, the five competency domains (emotional intelligence and self‐regulation, respectful and effective communication, ethical conduct and integrity, collaboration and empowerment, and relationship building and professional growth) collectively explain the mechanisms through which leaders may cultivate healthy workplace cultures. This multilevel perspective complements contemporary theories of authentic leadership [13, 14], transformational leadership [22], resonant leadership [15], psychological safety [7], and organizational well‐being [19], while extending these perspectives through an integrative framework that explains how civility functions as the mechanism linking leadership competencies to HWEs.

Unlike leadership models that emphasize either individual leader characteristics or organizational influence, the LCF explicitly links these dimensions through intentional leadership practices. This distinction has important implications because organizational cultures do not emerge spontaneously; they develop through repeated interactions, behavioral expectations, and leadership modeling. Leaders therefore shape workplace norms not only through formal authority but also through everyday behaviors that communicate respect, fairness, inclusion, accountability, and concern for others.

Existing leadership theories emphasize complementary dimensions of effective leadership but do not fully explain how leader character, relational influence, and transformational actions function together to cultivate healthy organizational cultures. The LCF synthesizes these perspectives by proposing that civility functions as the integrative mechanism through which leaders’ personal values and competencies are enacted into intentional leadership practices that shape organizational culture. This integration advances leadership scholarship by positioning civility as a strategic leadership capability that enables leaders to intentionally cultivate HWEs through observable leadership competencies and behaviors.

## 9. Theoretical Contributions

The LCF contributes to civility and leadership scholarship in three ways. First, it distinguishes leadership civility from civility leadership. Leadership civility represents the leader’s character, values, and competencies, whereas civility leadership reflects the intentional application of those capacities through behaviors that influence individuals, teams, and organizations. The five competency domains provide the developmental and behavioral bridge between these constructs.

Second, the LCF integrates authentic, resonant, and transformational leadership perspectives to explain how internal leadership capacities are translated into relational influence and strategic organizational action. Whereas existing leadership theories emphasize distinct aspects of leader character, relationships, or organizational influence, the LCF connects these dimensions through civility and specifies how they collectively contribute to HWEs.

Third, the LCF extends emerging civility‐focused leadership scholarship by specifying the competencies underlying intentional civility leadership. Ota et al. [23] identified four practices through which nurse leaders create civility including acknowledge, communicate, teach, and support through a constructivist grounded theory study of nurse leaders in Australian regional and rural settings. The LCF complements this practice‐focused perspective by conceptualizing how leadership competencies are organized and translated into intentional civility leadership and, ultimately, HWEs. Thus, the LCF provides a theoretical pathway linking who leaders are, the competencies they develop, what they intentionally do, and the environments they help create.

## 10. Practical Applications

The LCF may provide a conceptual foundation for nurse leaders seeking to intentionally cultivate HWEs across academic, clinical, and healthcare organizations. Because the framework identifies observable leadership competencies that may support healthy organizational cultures, it may inform organizational culture initiatives and educational strategies focused on civility. However, its use for leader selection, formal performance evaluation, or other high‐stakes personnel decisions should await empirical validation of the framework and associated measures.

The framework may also provide a conceptual basis for organizational initiatives that strengthen psychological safety, employee engagement, teamwork, and organizational health. Potential applications include leadership orientation, shared governance initiatives, and organizational culture initiatives. These applications should be considered developmental rather than evaluative until the framework and associated measures have been empirically validated.

Rather than viewing civility as an isolated intervention or communication strategy, the LCF positions civility as a strategic leadership capability that may contribute to employee well‐being, collaboration, patient safety, and organizational effectiveness [8]. These potential outcomes represent theoretical propositions of the framework and require empirical investigation. Although developed within nursing, the LCF may have relevance to other complex organizational settings; however, its applicability beyond nursing and healthcare should be examined through future research rather than assumed.

## 11. Leadership Development

The LCF provides a theoretical foundation for competency‐based leadership development. Leadership competencies such as emotional intelligence, respectful communication, ethical decision‐making, collaboration, and relationship building can be intentionally developed through coaching, mentoring, reflective practice, simulation‐based education, and other experiential learning strategies [15].

These competencies may be strengthened through repeated practice, feedback, and reflection, enabling leaders to translate civility competencies into intentional leadership behaviors. Individual leader development strategies may include simulation‐based leadership scenarios, civility education sessions, structured reflection following challenging interactions, peer coaching, mentoring relationships, executive coaching, conflict management workshops, and facilitated debriefings after complex clinical or organizational events.

Leadership development informed by the LCF should emphasize both personal growth and behavioral application. Developing self‐awareness, emotional regulation, and ethical judgment establishes the internal foundation of leadership civility, whereas structured opportunities for practice, reflection, coaching, and feedback may enable leaders to translate these competencies into civility leadership. Future research should examine whether leadership development approaches informed by the LCF strengthen civility leadership and contribute to healthier organizational environments.

## 12. Limitations

As a conceptual framework, the LCF has not yet been empirically tested. The proposed relationships should therefore be considered theoretically derived and require validation across diverse organizational settings, leadership roles, and cultural contexts. Future research should examine the framework’s construct validity, predictive validity, and practical utility and evaluate the relationships among the five leadership competency domains, leadership civility, civility leadership, and HWE outcomes. Empirical studies are also needed to determine the framework’s applicability across healthcare disciplines and other organizational sectors.

## 13. Future Directions

The LCF preceded development of the Leadership Civility Index (LCI). The five competency domains were derived conceptually through the integrative synthesis underlying the LCF and were not selected based on the LCI. The LCI was subsequently developed as a leadership assessment instrument informed by workplace civility and leadership scholarship, psychological safety, emotional intelligence, HWE literature, and the original Clark Workplace Civility Index [10]. Although the LCI aligns with the five LCF competency domains, its items also address cross‐cutting constructs, including psychological safety, inclusion, accountability, conflict management, and organizational stewardship. Thus, the LCF provides theoretical structure, whereas the LCI provides a potential measurement resource for examining selected framework constructs. Psychometric evaluation of the LCI is ongoing and will help determine its dimensional structure, measurement properties, and utility for future empirical testing of the LCF.

Future research should empirically test the relationships proposed within the LCF, including associations among leadership civility, the five competency domains, civility leadership, psychological safety, trust, inclusion, collaboration, engagement, and HWEs. Research should also examine whether these relationships extend to organizational outcomes such as employee well‐being, retention, patient safety, and organizational effectiveness. These outcomes represent theoretical propositions of the framework rather than established effects and should be evaluated through appropriately designed empirical and longitudinal studies.

Although developed primarily within nursing and healthcare, the LCF is bounded by its current conceptual and empirical foundation in these settings. Its applicability beyond healthcare should therefore be examined through future research rather than assumed. Future research should also examine the framework’s explanatory and predictive utility across diverse healthcare settings and organizational contexts. Future investigations should evaluate interventions designed to strengthen leadership civility competencies, including executive coaching, leadership development programs, simulation‐based learning, reflective practice, and organizational culture initiatives. Cross‐cultural validation and implementation studies will further clarify how the framework functions across diverse healthcare systems, organizational cultures, and leadership contexts.

## 14. Conclusion

The LCF provides an integrative conceptual model for explaining how leadership civility may be translated into intentional civility leadership through five interdependent competency domains. By integrating authentic, resonant, and transformational leadership perspectives, the framework offers a theoretically grounded structure for examining how leaders’ internal capacities and intentional behaviors may contribute to psychological safety, trust, inclusion, collaboration, engagement, and HWEs. The LCF is conceptual and has not yet been empirically tested. Its proposed relationships, including potential associations with employee well‐being, retention, patient safety, and organizational effectiveness, therefore remain theoretical propositions for future investigation rather than established effects. Empirical testing is needed to establish the framework’s construct validity, explanatory utility, and practical utility before it is applied to high‐stakes leadership selection or performance evaluation.

## Funding

No funding was received for this manuscript.

## Conflicts of Interest

Cynthia M. Clark is the developer of the Leadership Civility Index (LCI) and holds intellectual property rights associated with the instrument. The LCI is copyrighted/licensed through Boise State University. This relationship is disclosed in accordance with journal policy.

## Data Availability

Data sharing is not applicable to this article as no datasets were generated or analyzed during the current study.
